# Cancer as a System Failure

**Published:** 2007-04-03

**Authors:** Dennis Wigle, Igor Jurisica

**Affiliations:** 1 Division of Thoracic Surgery, Mayo Clinic Cancer Center, 200 First St. SW Rochester, Minnesota 55905, U.S.A; 2 Ontario Cancer Institute, PMH/UHN, Toronto Medical Discovery Tower, Division of Signaling Biology, Life Sciences Discovery Centre, 101 College Street, Toronto, Ontario M5G 1L7

Decades of focused cancer research have demonstrated the oncogenic process to be frustratingly complex. Despite many triumphs in scientific and clinical understanding, we still do not understand the formation of most solid tumors at a basic level. Each newly discovered molecular signature or profile brings to attention several exceptions in the form of mutations or histological subtypes that significantly change the applicability of the new knowledge to clinical practice. This has hampered improvements in detection, diagnosis, and treatment strategies.

Most solid tumors arise from a spectrum of genetic, epigenetic, and chromosomal alterations. The volume of such observations from both patient samples and tumor models in the cancer literature is overwhelming. Despite this, the variations in the molecular alterations that can give rise to cancer can be broadly grouped into a handful of traits that cancer cells must acquire for malignant transformation to occur. The original description by Hanahan and Weinberg of the “hallmarks” of cancer remains a seminal description that looks beyond the detailed molecular discoveries governing malignant transformation, and integrates them into a conceptual framework underlying all cancers (Hanahan and Weinberg, 2000). This framework simply but insightfully states that molecular alterations can be classified by dysfunction in as many as six different regulatory systems that must be perturbed for a normal cell to become cancerous (Khalil and Hill, 2005). These include many diverse and seemingly non overlapping biological processes, including (1) self-sufficiency in growth signals, (2) insensitivity to anti-growth signals, (3) evasion of apoptosis, (4) limitless replicative potential, (5) sustained angiogenesis, and (6) tissue invasion and metastasis. They define genetic instability as an “enabling characteristic” that facilitates the acquisition of other mutations due to defects in the repair of DNA. Although some cancer subtypes are defined by a single genetic alteration leading to a primary defect in one of the above listed processes, most solid tumors responsible for the largest burden of human illness are heterogeneous lesions characterized by many if not all defects observable simultaneously. This includes lung, breast, prostate, colon, and central nervous system tumors among others. In our attempts to understand tumorigenesis by reductionism, much work has gone into the study of individual biologic processes referred to as the “hallmarks” of cancer. Increased understanding for many of these biologic modules has unfortunately not generated parallel understanding of the root cause of cancers and how best to treat them.

The concept of cancer as a system failure and the potential to use systems biology approaches to understand the disease is generating significant discussion in the literature as investigators grapple with how to do this (Kitano, 2002; Alberghina, Chiaradonna et al. 2004; Spencer, Berryman et al. 2004; Khalil and Hill, 2005; Hornberg, Bruggeman et al. 2006). The mere recognition of cancer as a systems biology disease is a key first step. This hypothesis views the individual defects observable in solid tumors cumulatively as system failures either at the cellular or multicellular level. A systematic study and understanding of oncogenic network rewiring (Pawson and Warner, 2007) opens the potential to use systems biology approaches to generate testable models of different tumors, an exciting and as of yet unexplored realm of cancer biology.

System level understanding, the approach advocated in systems biology, requires a change in our notion of “what to look for” in biology (Kitano, 2002). While an understanding role of individual genes and proteins continues to be important, the focus is superseded by the goal of understanding a systems structure, function and dynamics. This evolution in thought in the life sciences has produced a profound transformation at the threshold of what is widely regarded as the century of biology (Kafatos and Eisner, 2004). From a collection of narrow, well-defined, almost parochial disciplines, they are rapidly morphing into domains that span the realm of molecular structure and function through to the application of this knowledge to clinical medicine. The results of teams of individual specialists dedicated to specific biological goals are providing insight into system structures and function not conceivable a decade ago.

Identifying all the genes and proteins in an organism is analogous to creating a list of all parts of a complex device or machine, such as an airplane. While such a list provides a catalog of individual components, it alone is not sufficient to understand the complexity underlying the engineered object (Kitano, 2002). One cannot readily build an airplane or understand its functional intricacies from a parts list alone. One needs to understand how these parts are assembled to form the structure of an airplane. This is biologically analogous to drawing an exhaustive diagram of gene-regulatory interactions and their biochemical interactions, as these diagrams would provide a limited knowledge of how changes in one part of the system may affect other parts. To understand how a particular system functions, how the individual components interact during operation and under failure must be examined first. From an engineering perspective, answers to key questions become critical, such as, what is the voltage on each signal line? How are the signals encoded? How is the voltage stabilized against noise and external fluctuations? How do the circuits react when a malfunction occurs in the system? What are the design principles and possible circuit patterns, and how can they be modified to improve system performance? (Kitano, 2002)

Why has systems biology received so much recent attention? In short, it is because the key first step of defining system structures has quickly advanced from fantasy to reality in the post-genomic era. The achievement of full genome sequencing projects in many organisms, including *Homo sapiens*, has defined the “parts list” for growth, development, and normal physiologic function. The technological development associated with these achievements has spawned the nascent fields of genomics, proteomics, and multiple ”-omic” disciplines defined by their systematic, data-driven approaches to biological experimentation. These approaches are increasingly being applied to the question of understanding cancer.

The volume of data generated by multiple high-throughput platforms has outpaced the computational and mathematical models to integrate this information for advances in true biologic understanding (Figure 1). This will continue to be a bottleneck for the near future.

We review a number of relevant observations in the context of the “hallmarks” of cancer and discuss the issue of data integration in performing systems-level experiments.

## Angiogenesis

One line of support for cancer as a system failure is the evidence for the angiogenic phase of tumorigenesis being a critical component of tumor progression once lesions reach a certain size. A tumor cannot typically grow beyond a volume of approximately 10^6^ cells without neo-angiogenesis taking place to supply blood to the tumor (Folkman, 1990). In this framework, the acquisition of mutations due to genetic instability leads to the transformation of normal cells in our body into cancer cells. This phase is not inherently lethal, and generally results in a microscopic tumor. The second phase involves a switch to the angiogenic phenotype, involving the constant recruitment of new blood vessels to the tumor. This converts the nonlethal *in situ* lesions into an expanding mass that is potentially lethal. A number of critical factors have been identified with the capacity to launch angiogenesis and enter a lethal phase of cancer, including basic FGF, VEGF, PDGF, thrombospondin, tumstatin, canstatin, endostatin, and angiostatin. Interestingly, there is a very low incidence of solid tumors in patients with Down Syndrome, who circulate elevated levels of endostatin, an endogenous angiogenesis inhibitor, due to an extra copy of chromosome 21. Tumor progression clearly depends on the balance between the *in situ* tumor’s total angiogenic output and an individual’s total angiogenic defense (Folkman, 1990).

In a similar vein, the balances holding an *in situ* lesion in check before it advances to a focal tumor involve a balance between a high rate of tumor cell division and cell death. A number of immune surveillance mechanisms are postulated to be involved in such a balance. For example, autopsies of individuals dying of trauma often reveal microscopic colonies of cancer cells, also known as *in situ* tumors. Virtually all autopsied individuals aged 50 to 70 have *in situ* carcinomas in their thyroid gland, whereas only 0.1% of individuals in this age group are diagnosed with thyroid cancer during this period of their life. It has long puzzled clinicians and scientists why this cancer develops and progresses to be lethal only in a very small percentage of people. The realization that a lot of us carry *in situ* tumors, but do not develop the disease, suggests that these microscopic tumors are mostly dormant, and need additional signals to grow (Folkman, 1990).

## Aberrant Cell Signaling

As we learn more and more about the signal transduction pathways governing cell growth and division, it has become clear that the majority are not linear pathways acting in isolation. A large amount of cross-talk exists within and between pathways. Individual components have the potential to act in multiple pathways, creating unexpected events when mutated. How these networks and their regulatory constraints are controlled is a significant research challenge.

One of the prominent pathways mutated in many tumor types is the EGFR-ras-MAPK pathway. Epidermal growth factor receptor (EGFR) was identified as a candidate for therapeutic control of cancer more than two decades ago. It is expressed in most patients with non-small cell lung cancer (NSCLC), and has a role in cellular proliferation, inhibition of apoptosis, angiogenesis, metastatic potential, and chemoresistance (Blackhall, Ranson et al. 2006). Activation of the EGFR pathway is able to promote tumor growth and progression, stimulating cancer cell proliferation, production of angiogenic factors, invasion and metastasis, and inhibiting apoptosis.

Ras mutations occur in approximately 30% of lung adenocarcinomas, with some data to suggest that prognosis can be influenced by the presence or absence of a ras mutation. Mouse models of lung cancer harboring germline activating ras mutations develop lethal lung adenocarcinoma. Tumors from these animals develop a number of subsequent genetic alterations as a downstream consequence of ras activation. However, many questions remain unanswered; specifically the immediate downstream molecular events associated with aberrant ras signaling. Why germline activating ras mutations produce predominantly lung cancer suggests some element of tissue context to ras activation that remains to be discovered.

**Figure 2.**
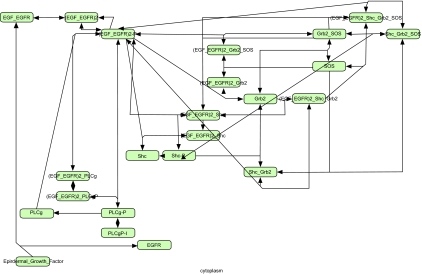
EGFR signaling pathway (Oda, Matsuoka et al. 2005) from EMBL-EBI Systems Biology Markup Language curated model repository ((Le Novere, Bornstein et al. 2006); http://www.ebi.ac.uk/compneur-srv/biomodels-main/publ-models.do?cmd=MODELS:SRT&sof=nam&sod=asc#models), visualized using CellDesigner 3.5.1 (http://www.systems-biology.org/cd/).

**Figure 3.**
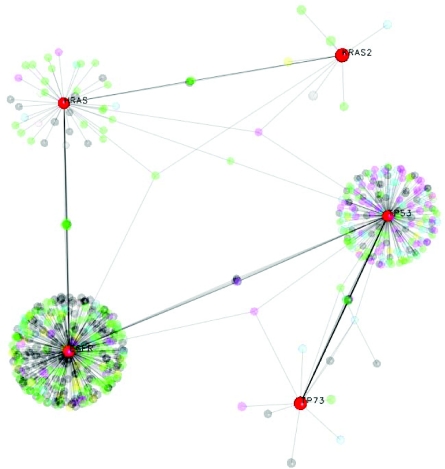
EGFR—RAS protein interaction network from OPHID (Brown and Jurisica, 2005), visualized in NAViGaTor ver. 1.1 (http://ophid.utoronto.ca/navigator) in a 3D mode. Although EGFR, hRAS, kRAS and p53 are not directly linked, these major hubs in the network are highly mutually interconnected. Nodes in the graph are proteins, while edges correspond to interactions. Node color corresponds to GeneOntology biological function, except for highlighted EGFR, p53, p73, hRAS and kRAS. To reduce graph complexity, nodes except for highlighted ones, and related edges are partially translucent using alpha-blending option in NAViGaTor.

The protein-protein interaction network surrounding EGFR—RAS signaling contains a number of well-characterized proteins, as shown on an example from the EBI SMBL Model Repository (Oda, Matsuoka et al. 2005; Le Novere, Bornstein et al. 2006) in Figure 2, and visualized in CellDesigner 3.5.1 (http://www.systems-biology.org/cd/). Information from protein-protein interaction databases, such as OPHID/I^2^D ((Brown and Jurisica, 2005); http://ophid.utoronto.ca/i2d), further extends the potential to study and model these pathways under specific stimuli or in different tissues. Figure 3 shows one such visualization in NAViGaTor 1.1 (http://ophid.utoronto.ca/navigator), highlighting only core proteins, and suppressing other details by using alpha-blending. Individual nodes in the graph represent proteins, while edges correspond to known and predicted interactions. The color of individual nodes (except for red-highlighted ones) denotes different GeneOntology biological functions.

In non-small cell lung cancer (NSCLC), the initial studies of epidermal growth factor receptor (EGFR) tyrosine kinase inhibitors (TKIs) brought significant enthusiasm for targeted therapeutic approaches. Initial studies demonstrated that EGFR inhibition could lead to dramatic tumor regression in 10% to 15% of all treated patients. However, not all patients seemed to benefit from this treatment. A careful examination of patients who benefited from single-agent EGFR TKIs in phase II clinical trials, including unselected patients and those treated in the AstraZeneca gefitinib expanded access program, revealed clinical characteristics associated with an increased likelihood of a clinical or radiographic response. Patients most likely to achieve a radiographic response to EGFR TKIs were women, lifetime non-smokers, patients with adenocarcinomas, and those of Japanese ethnicity.

In the spring of 2004, two simultaneously published studies examined case series of patients who had had dramatic clinical and/or radiographic responses to gefitinib (Lynch, Bell et al. 2004; Paez, Janne et al. 2004). Thirteen of 14 patients were found to have somatic activating mutations in the EGFR kinase domain, whereas none of the 11 patients who progressed on gefitinib had these EGFR mutations. Subsequently, EGFR mutations have been investigated in several series of NSCLC tumors from surgically resected patients or in patients treated with gefitinib or erlotinib. The mutation frequency appears to vary based on different patient characteristics, but very much mirrors the clinically defined subgroups deemed likely to achieve radiographic responses to EGFR TKIs. EGFR mutations are typically found in the first four exons of the tyrosine kinase domain of EGFR. Three types of mutations have been described: (1) deletions in exon 19 account for about 60% of all mutations; (2) a common missense mutation in exon 21 (L858R) accounts for another 25%; and, finally, (3) rare point mutations in exons 18, 20, and 21 and insertion/duplications in exon 20 account for the remainder (Johnson and Janne, 2005).

One of the startling aspects of the Paez et al. paper was that despite sequencing of the exons encoding the activation loops of 47 of the 58 human receptor tyrosine kinase genes in the human genome in 58 NSCLC samples, only 3 of the tumors, all lung adenocarcinomas, showed heterozygous missense mutations in EGFR not present in the DNA from normal lung tissue from the same patients (Paez, Janne et al. 2004). No mutations were detected in amplicons from other receptor tyrosine kinase genes. All three tumors had the same EGFR mutation, predicted to change leucine-858 to arginine. Why EGFR is the sole RTK mutated in NSCLC is surprising and points to the important role of the receptor and its signaling axis.

The EGFR TKI example demonstrates the potential for targeted agents directed in a personalized manner using molecular substaging. Clearly, this is only the tip of the iceberg for targeted therapies in NSCLC. Integration of these with other agents targeting different pathways may herald the age of multi-targeted small-molecule inhibitors that may come to supercede selective mono-targeted agents (Blackhall, Ranson et al. 2006).

## DNA Damage and Repair

Normal growth and development is dependent on the accurate transmission of genetic information from one cell to its progeny. Faithful transmission requires not only accurate replication of DNA, but also the ability to survive spontaneous and induced DNA damage while minimizing the number of heritable mutations (Zhou and Elledge, 2000). To achieve this fidelity, mammalian systems have evolved sophisticated surveillance mechanisms that monitor the structure of chromosomes and coordinate repair and cell-cycle progression. The genome is constantly exposed to exogenous DNA damaging events in the form of radiation, viral infection and chemicals. Endogenous processes such as DNA replication and free radical formation also threaten the integrity of the genome. DNA damage is directly deleterious to cells, and if left unrepaired, plays a direct role in the initiation and progression of many tumor types (Zhou and Elledge, 2000).

There are multiple cellular mechanisms for correcting or repairing incorrect, damaged, or broken DNA sequences, but in mammalian cells, nucleotide excision repair is the major pathway for removing damaged bases from DNA. Much of our knowledge of the nucleotide excision repair process comes from studies of Xeroderma pigmentosum, in which inherited mutations of certain crucial nucleotide excision repair genes disable the repair of DNA damage from ultraviolet light, a defect that results in multiple skin cancers of various types in skin that has been exposed to the sun (Cleaver, 2005).

Platinum-based chemotherapeutic regimens have become the mainstay of treatment for many tumor types, particularly non-small cell lung cancer. Platinum compounds exert their cytotoxic effects by binding covalently to genomic DNA, forming adducts that result in altered forms of DNA. Such couplings activate the DNA-repair process, and unless these adducts are repaired before the DNA replicates, they can lead to nucleotide substitutions, deletions, and chromosome rearrangements that are propagated in daughter cells, or to activation of cell-signaling pathways that result in cell death via apoptosis.

Cisplatin molecules bind covalently to genomic DNA, forming a bulky, helix-distorting adduct. In chemosensitive cells with low nucleotide excision repair activity, apoptosis usually follows, while in chemoresistant cells with high nucleotide excision repair activity, the adduct may be excised and the DNA repaired. The adduct is first recognized, and proteins of the nucleotide excision repair complex are assembled at the adduct site. The heterodimeric protein excision repair cross-complementation group 1 (ERCC1)–XPF is the last component to be assembled and appears to be the rate-limiting step in this process. The excised segment is repaired by polymerases and the accessory replication proteins PCNA, RPA, and RFC. The integrity of the damaged strand is restored by DNA ligase. The protein ribonucleotide reductase M1 (RRM1), although not an integral part of the repair complex, catalyzes the biosynthesis of deoxyribonucleotide from the corresponding ribonucleotides, providing the building blocks for reconstitution of the excised oligonucleotide (Gazdar, 2007).

Two recent high-profile papers point to the clinical importance of these pathways. Olaussen et al. for the IALT Bio Investigators used immunohistochemical analysis to determine the expression of the excision repair cross-complementation group 1 (ERCC1) protein in operative specimens of NSCLC (Olaussen, Dunant et al. 2006). The patient cohort consisted of those enrolled in the International Adjuvant Lung Cancer Trial, thereby allowing a comparison of ERCC1 expression with the effect of adjuvant cisplatin-based chemotherapy on survival. Adjuvant cisplatin-based chemotherapy is known to improve survival among patients with completely resected NSCLC, but there is no validated clinical or biologic predictor for the potential benefit of chemotherapy. Among 761 tumors, ERCC1 expression was positive in 335 (44%) and negative in 426 (56%). A benefit from cisplatin-based adjuvant chemotherapy was associated with the absence of ERCC1. Adjuvant chemotherapy, as compared with observation, significantly prolonged survival among patients with ERCC1-negative tumors but not among patients with ERCC1-positive tumors. Among patients who did not receive adjuvant chemotherapy, those with ERCC1-positive tumors survived longer than those with ERCC1-negative tumors. The authors concluded that patients with completely resected NSCLC and ERCC1-negative tumors appear to benefit from adjuvant cisplatin-based chemotherapy, whereas patients with ERCC1-positive tumors did not.

In contrast, recent evidence suggests that RRM1, the regulatory subunit of ribonucleotide reductase, is involved in carcinogenesis, tumor progression, and the response of NSCLC to treatment. Using an automated quantitative determination of the RRM1 protein in routinely processed histologic specimens, Zheng et al. measured the expression of RRM1 and two other proteins relevant to NSCLC (Zheng, Chen et al. 2007). This included the ERCC1 and the phosphatase and tensin homologue (PTEN). The results were compared with the clinical outcomes in 187 patients with early-stage NSCLC who had received only surgical treatment. RRM1 expression correlated with the expression of ERCC1 but not with the expression of PTEN. The median disease-free survival exceeded 120 months in the group of patients with tumors that had high expression of RRM1 and was 54.5 months in the group with low expression of RRM1. The overall survival was more than 120 months for patients with tumors with high expression of RRM1 and 60.2 months for those with low expression of RRM1. Among these 187 patients, the survival advantage was limited to the 30% of patients with tumors that had a high expression of both RRM1 and ERCC1. The authors concluded that both RRM1 and ERCC1 are determinants of survival after surgical treatment of early stage NSCLC.

The paradox highlighted by these two papers is the possibility that the immunohistochemical presence of ERCC1 in conjunction with RRM1 may actually be beneficial in early stage NSCLC; however, this may not translate into benefit from receiving platinum-based adjuvant chemotherapy. In other words, translational utilization of ERCC1 as a NSCLC biomarker would suggest that its presence in early stage disease implies a good prognosis, whereas its absence in later stages, where platinum-based chemotherapy may be a treatment option, would provide a better prognosis.

## An Atlas of Cancer Genes

It has been suggested that 5–10% or more of the ~25,000 putative genes encoded in the human genome probably contribute to oncogenesis (Strausberg, Simpson et al. 2003). However, a recent exhaustive census based on an updated list from the Sanger Centre has compiled only 354 experimentally validated genes that are causally implicated in neoplasia development (Futreal, Coin et al. 2004). This accounts for only about 1% of all predicted human genes. These cancer genes have historically been identified in a step-wise manner by the positional cloning of individual familial susceptibility loci, the discovery of viral and mutated forms of cellular proto-oncogenes, or by the association of specific chromosome anomalies with gain- or loss-of-function alleles of select genes (Hu, Bader et al. 2007). This contrasts with the actual burden of disease, where solid tumors of unknown genetic aetiology account for most cancer cases—for example, lung, colon, breast, prostate and pancreatic tumors lead to ~55% of all cancer mortality in the United States based on statistics from the American Cancer Society, equating to 316,305 of 564,830 cancer deaths predicted for 2006.

**Figure 4.**
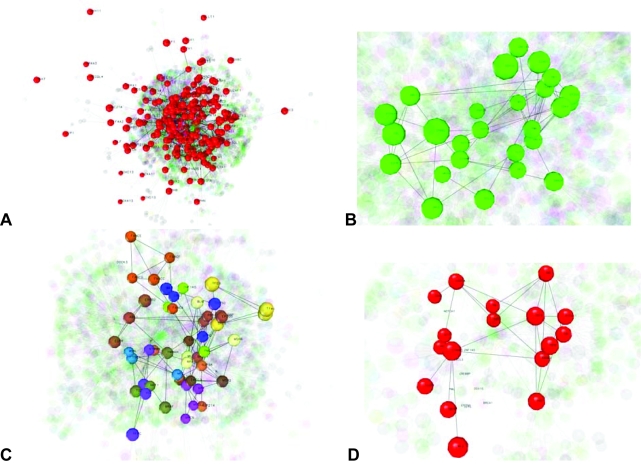
Mapping of 354 oncogenes (Futreal, Coin et al. 2004) to an OPHID database (Brown and Jurisica, 2005) to produce an oncogene protein-protein interaction network. Visualization is done in NAViGaTor 1.1. using a 3D mode (http://ophid.utoronto.ca/navigator). **A** Oncogene interaction network with oncogenes highlighted in red (202 of the 354 oncogenes had an interaction in OPHID). The resulting network comprises 2,411 proteins and 4,019 interactions. **B** Top 1% of hubs (highly connected proteins) in the network is highlighted in green (n = 26). **C** The oncogene interaction network contains 12 highly connected subgraphs, highlighted by different colours. **D** A small EGFR subnetwork with related top 1% hubs highlighted in red, generated by inducing the graph on EGFR and its immediate interacting partners.

A protein-protein interaction network for the cancer gene census demonstrates that most of these proteins are highly interconnected, as shown in Figure 4.

Comprehensive analyses of interaction networks has suggested that links between hub proteins are systematically suppressed (Maslov and Sneppen, 2002). Such networks would be theoretically more robust by reducing the likelihood of cross-talk between functional modules, which in turn would localize effects of deleterious perturbations. Interestingly, contrary to these earlier results, the “oncohubs” are highly interconnected, suggesting significant cross-talk between associated pathways. To what degree publication bias has influenced network topology is unclear. One hypothesis is that these onco-modules are heavily utilized for normal growth, development, and function; hence, heavily buffered against the effects of perturbation. Even exceptionally low error rates, however, have dramatic consequences in the context of solid tumor formation over time. We (*Homo sapiens*) were unfortunately not designed to live to 100 years of age problem-free.

## Integrative Cancer Informatics–A Systems Approach to Cancer

Despite the increasing introduction of novel and diverse chemotherapeutic agents, most cancers remain diseases with devastating mortality rates. The accumulation of data from systematic high-throughput experiments has brought the potential to construct models of how biological systems work at the cell and whole organism level. However, to ensure more accurate modeling of biological systems, we need to improve data integration, visualization, and intertwine biological experimentation with computational analysis.

To address complexities of systems level understanding of cancer, Deisboeck et al. motivate the need for an integrated infrastructure for tracking ongoing research related to modeling cancer research in a systematic way. A series of web interfaces are provided to illustrate the existing range of functionalities provided by the CViT (Center for the Development of a Virtual Tumor), which is one of the National Cancer Institute’s recently established Integrative Cancer Biology Programs. CViT focuses on: 1) tools for communal information exchange between cancer researchers; 2) a desktop environment linking users with integrated resources housed by the CViT centre; and 3) a repository for existing digital models of cancer.

Levine et al. 2006 tackle the problem of mathematical modeling of signal transduction, namely the formation of an avascular tumor based on the loss by gene mutation of the tumor suppressor function of p53. While the model is complex, there are still additional variables that could have important roles in the initiation of tumor growth. It is the intertwined biologic experimentation and computational modeling/analysis that will drive transformation of these initial models into more accurate and useful computational cancer models.

Reif et al. introduce an application of Exploratory Visual Analysis (EVA), a bioinformatics tool for exploring statistical analysis results rather than raw data in a visual manner. The main aim is to provide a simple, yet integrated view, and enable interpretation of p-values in the context of Gene Ontology, biochemical pathways, protein domains, chromosomal locations, or phenotypes. The authors provide an evaluation of EVA using publicly available microarray datasets of high-grade glioblastoma multiforme tumors and indolent, low-grade pilocytic astrocytomas.

Do et al. present a comparison of two clustering techniques: a mixture model based on an Expectation Maximization (EM) algorithm called EMMIX-GENE, and a GeneClust algorithm that uses the gene shaving method. The main focus is to diminish the problems in high dimensional domains—large number of variables and small number of samples. Both algorithms are evaluated on two, publicly available microarray datasets, by comparing measures of accuracy and speed.

Quayle et al. describe a computational approach for selecting sets of gene targets for cancer therapy. This approach involves building interaction networks for cancers and corresponding normal samples, and attempting to select a set of targets that cause a greater disruption in a cancer network compared to normal. Using both human curated and predicted protein interaction networks, the authors confirm the finding that curated data is highly biased towards known and disease-related targets. It would be useful to see how the results change by integrating with the larger human interactome, from curated, predicted, and high-throughput experiments, such as in the Interologous Interaction Database I^2^D (http://ophid.utoronto.ca/i2d).

## The future—Data integration to systems-level experiments

Integrating across multiple systems is a formidable challenge. Each area alone is extremely complex. However, as system structures are now being defined, the key first steps toward system level integration are becoming possible. Much work remains, however, prior to being able to feasibly study multiple system modules as a whole. The role of computational biology and mathematical modeling as an integral part of these advances is becoming increasingly clear. The next round of major advances will clearly arise from the combined efforts of integrated study groups with expertise in both computational and biologic experimentation.

## Figures and Tables

**Figure 1.**
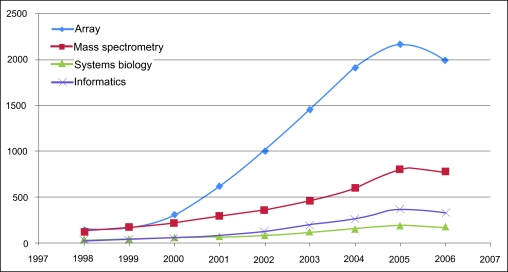
Estimating cancer research growth and utilization of diverse high-throughput platforms as measured by number of Medline references. The apparent decline in 2006 could be explained by not finalized references in Medline (February 9, 2007).
